# Closed circular genome sequence of a *Microcystis aeruginosa* PCC7806 Δ*mcyB* (UTK) non-toxic mutant

**DOI:** 10.1128/MRA.00700-23

**Published:** 2023-10-19

**Authors:** Gwendolyn F. Stark, Alexander R. Truchon, Elke Dittmann, Steven W. Wilhelm

**Affiliations:** 1 Department of Microbiology, University of Tennessee Knoxville, Knoxville, Tennessee, USA; 2 Department of Microbiology, Institute for Biochemistry and Biology, University of Potsdam, Potsdam, Germany; California State University San Marcos, San Marcos, California, USA

**Keywords:** cyanobacteria, harmful algal blooms, cyanotoxins, microcystin

## Abstract

Here we report the complete, closed genome of the non-toxic *Microcystis aeruginosa* PCC7806 Δ*mcyB* mutant strain. This genome is 5,103,923 bp long, with a GC content of 42.07%. Compared to the published wild-type genome (*Microcystis aeruginosa* PCC7806SL), there is evidence of accumulated mutations beyond the inserted chloramphenicol resistance marker.

## ANNOUNCEMENT

*Microcystis aeruginosa* PCC7806 Δ*mcyB* is a non-toxic strain available at the Pasteur Culture Collection (1). This mutant is widely used in comparative studies with the toxic wild-type strain PCC7806 (2 – 15). Except for a chloramphenicol cassette inserted in the *mcyB* gene, the mutant and wild-type genomes were thought to be identical, although this had not been confirmed. Whole-genome sequencing revealed that our strain, which has undergone live passage and cryopreservation (−150°C) for the last 7 years after an aliquot was transferred from Potsdam (Germany) to Knoxville (USA), has accumulated mutations relative to the published 7806SL genome—we have added the “UTK” annotation to signify our specific isolate. Of note, our mutant strain has an additional putative transposase gene (*tpnA*), not seen in the 7806SL wild type, inserted in an SLC13-family permease gene. There is also a region of the genome that assembled in a manner suggesting a large inversion (~2.5 Mbp) has occurred. This paper serves as a cautionary tale for labs to routinely re-sequence strains.

Axenic cultures of *Microcystis aeruginosa* PCC7806 *ΔmcyB* (UTK) were maintained in 50 mL of modified CT medium (B-glycerophosphate was replaced with 1 mL of Na_2_HPO_4_ (23.19 mg/mL) and grown under a 14-h/10-h day/night cycle (~35 µmol photons/m^2^/s). Late-log phase cells were pelleted (15,000 × *g*, 10 min) and resuspended in 400 µL TE buffer. RNase (0.6 µL of 10 mg/mL) and 120 µL of lysozyme (20 mg/mL) were added, and cells were incubated at 37°C for 30 min. Then, 6 µL of proteinase K (20 mg/mL) in 3 mM CaCl_2_ and 200 mM Tris buffer and 39.5 µL of 20% SDS were added before incubation at 55° C for 2 h. DNA was extracted via a phenol-chloroform method (16). Half the extracted DNA was used for long-read sequencing (DNA was not sheared), and the other half was used for Illumina short-read sequencing. Long reads were sequenced in-house using a MinION R9.4.1 flow cell (Oxford Nanopore Technologies). Library prep for high-molecular weight DNA was prepared with a ligation sequencing kit, SQK-LSK110, per manufacturer protocols, which enriched for reads >3 kb, but no formal size selection was performed (Oxford Nanopore Technologies). Long-read sequencing resulted in 320,921 raw reads (N_50_ = 17.55 kb). For Illumina sequencing, DNA was sent to SeqCenter (Pittsburgh, PA). Sample libraries were prepared using an Illumina DNA prep kit and IDT’s 10-bp unique dual indices and were processed on an Illumina NextSeq 2000, generating 8,278,500 151-bp paired-end reads. Demultiplexing, quality control, and adapter trimming were performed with bcl-convert using default parameters (v.3.9.3) (Illumina) (Table 1).

**TABLE 1:** Genomic comparison of PCC7806 Δ*mcyB-*UTK to the published annotated genome 7806SL^*a*^

	*Microcystis aeruginosa* PCC7806 Δ*mcyB-*UTK	*Microcystis aeruginosa* PCC7806SL
Genome size (total bp)	5,103,923	5,139,339
GC content (%)	42.07	42.09
*tnpA* transposons	16	15

^*a*^ We note the version of Prokaryote Genome Annotation Pipeline (PGAP) we used to annotate PCC7806 Δ*mcyB-*UTK failed to annotate one copy of the 243-bp *tnpA* gene that was annotated in the PCC7806SL published genome: we confirmed its presence manually.

For genome assembly, default parameters were used for all software. Bases were called using Guppy (v.6.01) (17), with the dna_r9.4.1_450bps_fast.cfg config file, and adapter sequences were trimmed using Porechop (v.0.2.4) (18), then filtered using NanoFilt (v.2.8.0) at a quality of 9 and a length of 500 (19). Illumina reads were trimmed using CLC Genomics Workbench (v. 21.0.4). Genome assembly using long and short reads was performed *de novo* using Unicycler (v.0.4.9b), using a predicted length of 5 Mbp (20). A circular contig was assembled, circularized, trimmed, and rotated by Unicycler. The final genome assembly was annotated using National Center for Biotechnology Information PGAP (annotation software revision: 2022–12-13.build6469), and genomic GC content was determined with QUAST (v.4.4)(21).

## Data Availability

All sequencing data are available on National Center for Biotechnology Information under the BioProject ID PRJNA995633. Raw reads can be found at the Sequence Read Archive (SRA) under accession numbers SRX21194812 (Nanopore) and SRX21194811(Illumina). The annotated genome assembly can be found under GCA_030553035.1.
